# Gravity Cues Embedded in the Kinematics of Human Motion Are Detected in Form-from-Motion Areas of the Visual System and in Motor-Related Areas

**DOI:** 10.3389/fpsyg.2017.01396

**Published:** 2017-08-17

**Authors:** Fabien Cignetti, Pierre-Yves Chabeauti, Jasmine Menant, Jean-Luc J. J. Anton, Christina Schmitz, Marianne Vaugoyeau, Christine Assaiante

**Affiliations:** ^1^Aix-Marseille Université, CNRS, LNC, Laboratoire de Neurosciences Cognitives Marseille, France; ^2^Aix-Marseille Université, CNRS, Fédération 3C Marseille, France; ^3^Prince of Wales Medical Research Institute, School of Public Health and Community Medicine, University of New South Wales, Randwick NSW, Australia; ^4^Aix-Marseille Université, CNRS, INT UMR 7289, Centre IRM Fonctionnelle Marseille, France; ^5^Lyon Neuroscience Research Center, Brain Dynamics and Cognition Team, CRNL, INSERM U1028, CNRS UMR 5292 Lyon, France; ^6^University Lyon 1 Lyon, France

**Keywords:** biological motion, gravity, functional MRI, motor resonance, form-from-motion perception

## Abstract

The present study investigated the cortical areas engaged in the perception of graviceptive information embedded in biological motion (BM). To this end, functional magnetic resonance imaging was used to assess the cortical areas active during the observation of human movements performed under normogravity and microgravity (parabolic flight). Movements were defined by motion cues alone using point-light displays. We found that gravity modulated the activation of a restricted set of regions of the network subtending BM perception, including form-from-motion areas of the visual system (kinetic occipital region, lingual gyrus, cuneus) and motor-related areas (primary motor and somatosensory cortices). These findings suggest that compliance of observed movements with normal gravity was carried out by mapping them onto the observer’s motor system and by extracting their overall form from local motion of the moving light points. We propose that judgment on graviceptive information embedded in BM can be established based on motor resonance and visual familiarity mechanisms and not necessarily by accessing the internal model of gravitational motion stored in the vestibular cortex.

## Introduction

Earth’s gravity is an important factor that influences visual perception. Psychophysical experiments demonstrated that several spatiotemporal characteristics of a visual scene are estimated employing implicit knowledge about the effects of gravity on moving objects in the physical world. For instance, visual gravity cues contribute to the perception of size, distance and flight time of falling objects (Watson et al., 1992; Huber and Krist, 2004; Brouwer et al., 2006; Moscatelli and Lacquaniti, 2011). Motion naturalness of a freely swinging pendulum is also established judging violations of the natural relation between period and length imposed by gravitational acceleration (Pittenger, 1990). Furthermore, manual interception of falling objects under microgravity is not accurately timed given the lack of object acceleration (McIntyre et al., 2001; Senot et al., 2012). Based on the fact that the visual system is quite poor at estimating image accelerations (Werkhoven et al., 1992), the above predictive behaviors in visual perception and interceptive responses involving knowledge about gravity were in favor of the existence of an internal model of gravitational motion internalized in the human brain. Functional magnetic resonance imaging (fMRI) experiments tested this hypothesis and demonstrated that an internal model implemented within a vestibular neural network, including the posterior insula, the retroinsular cortex and the temporoparietal junction, transforms the gravity vector into an abstract representation accessible by the visual system to establish judgments on gravitational motion of objects (Indovina et al., 2005; Lacquaniti et al., 2013). Likewise, Indovina et al. (2013) showed that a similar network is engaged during vertical self-motion coherent with natural gravity.

There is also evidence that gravity cues are critical for the visual perception of biological motion (BM) as presented with point-light displays (Jokisch and Troje, 2003; Shipley, 2003; Troje and Westhoff, 2006). Such displays, first described by Johansson (1973), convey a vivid impression of figures in motion, which is already decoded by young infants (Fox and McDaniel, 1982). The rudimentary information contained in point-light displays of BM is sufficient even to solve sophisticated recognition tasks, including identity and gender recognition (Cutting and Kozlowski, 1977; Kozlowski and Cutting, 1977; Pollick et al., 2005), emotion recognition (Pollick et al., 2001), and understanding of social interactions (Centelles et al., 2011). Interestingly, the detection and recognition of BM from point-light displays are disrupted once they are turned upside down (Sumi, 1984; Pavlova and Sokolov, 2000). An explanation for this ‘inversion effect’ would be that the novel orientation of the display makes the form of the stimuli unfamiliar so that individuals are no longer able to extract form and then determine the action (Reed et al., 2003). Nevertheless, it has been demonstrated that even when form information is disrupted, perception of the displays is still subjected to an inversion effect, which in turn likely results from the violation of the familiar (earth-based) spatiotemporal relations between body joints specified by the kinematics (Shipley, 2003; Troje and Westhoff, 2006). Therefore, the visual perception of BM from point light displays involves picking-up dynamic information from the kinematics of body movements that relies on the natural gravity.

At the brain level, a sensitivity to the inversion effect (as obtained by contrasting intact and inverted point-light displays) was found in several regions belonging to the BM perception network (Saygin et al., 2004), especially the occipito-temporal cortex and regions in the parietal (i.e., intraparietal sulcus) and frontal (i.e., caudal part of the middle/inferior frontal gyrus) lobes (Grezes et al., 2001; Grossman and Blake, 2001; Pavlova et al., 2004; Peuskens et al., 2005). With respect to the occipito-temporal cortex, data from Maffei et al. (2015) suggest that activity changes induced by displays with a non-normal gravitational kinematics is related to backward modulatory influences from regions that internalize the effects of Earth gravity on visual motion in general, namely the insula and the temporoparietal junction (Indovina et al., 2005, 2013; Lacquaniti et al., 2013). Thus, modulation of activity in the occipito-temporal cortex would signal mismatches (errors) between incoming and expected stimuli as predicted by the internal model of gravity stored in the vestibular cortex, meaning that predictive coding of gravity effects contributes to BM interpretation. However, there is no evidence that activation gradients in the previously mentioned posterior parietal and frontal regions when inverting BM displays constitute prediction errors that relate to the internal model of gravitational motion. It can only be argued that these regions are commonly involved during the execution and the observation of movements (Rizzolatti and Craighero, 2004; Dinstein et al., 2007; Kilner et al., 2007; Chong et al., 2008; Kilner et al., 2009; Saygin et al., 2012), or otherwise are core nodes of the mirror-neuron system (MNS) whose activation is often interpreted within the framework of motor resonance, whereby an observed action is understood through mapping onto the observer’s own motor representation. In this framework, interpreting gravitational cues embedded in BM would rely on a mechanism that ‘judges’ the compliance of the observed BM with naturalistic (Earth-based) BM stored in the observer sensorimotor repertoire, that is a sort of implicit coding of gravity effects that may not require a predictive code from the internal model of gravity.

Relevant to this explanatory framework is the fact that activity within the MNS was found to be sensitive to human kinematic invariants during action/motion observation (Dayan et al., 2007; Casile et al., 2010). In particular, these experiments reported that compliance of the moving stimuli with a natural law of motion (i.e., the two-thirds power law) was reflected in stronger activation in certain areas of the MNS, especially motor-related areas (e.g., ventral premotor cortex). Therefore, the more plausible the kinematics of the observed action, the stronger the resonance of the MNS. Alternatively, studies also examined the hypothesis that regions of the MNS, including motor-related and parietal areas, should not be active during the observation of biomechanically impossible movements that are not part of the observer’s motor repertoire (Stevens et al., 2000; Costantini et al., 2005). Although findings by Stevens et al. (2000) suggest that motor and parietal cortices are selectively activated to process movement that conforms to the capabilities of the observer, Costantini et al. (2005) showed that premotor areas coded movement regardless of whether it is biologically possible or impossible while parietal areas coded for movement plausibility (i.e., an activation gradient between possible and impossible movements). Overall, despite certain discrepancies, all these studies tend to demonstrate that violations of the physical laws that apply on Earth in displayed movements is inferred using motor resonance, with the sensory inputs being mapped onto one’s own body motor repertoire and thus coding the possibility of actually performing the same movements.

The present fMRI study investigated the cortical regions responsible for detecting graviceptive information during visual perception of BM. Gravity cues were manipulated by presenting point-light displays depicting a person moving under normogravity or microgravity, the displays having been recorded during parabolic flights (see Materials and Methods for details). The displayed avatars executed the same movements in both normogravity and microgravity so that shape characteristics changed only marginally between the two conditions but with different kinematic characteristics. We expected that coding of gravitational content in BM displays engages motor resonance, which should be reflected in a larger activity in regions of the MNS (i.e., a larger motor resonance) for normogravity BM displays given the closer match between the observed action and the observers’ own sensorimotor representations.

## Materials and Methods

### Participants

Twenty healthy right-handed volunteers (mean ± SD [range] age: 36 ± 8 [27–45] years; 9 females) participated in the study. All the participants were naïve as to the purpose of the study and never experienced microgravity. This study was reviewed and approved by the local Ethics Committee “CPP Sud-Méditerranée 1.” Before the study, all participants provided written informed consent. This study was carried out in accordance with the Declaration of Helsinki.

### Stimuli

**FIGURE 1:**
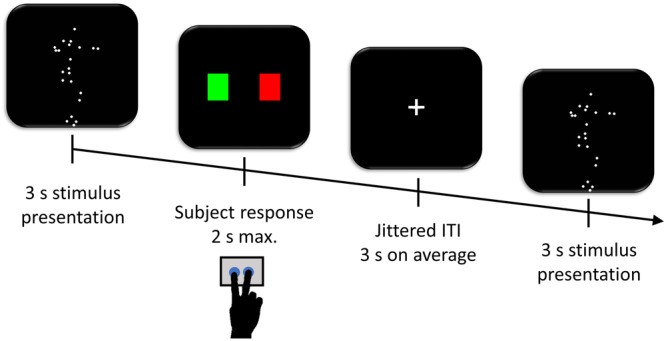
Task procedure. Participants observed the display (either normogravity or microgravity) on the screen for 3 s and had 2 s afterward to indicate whether the movement was performed on Earth (green rectangle) or in Space (red rectangle). Trials were separated by a jittered inter-trial interval (ITI) with a mean duration of 3 s during which a fixation cross was displayed at the center of the screen.

The stimuli were three-second silent point-light displays of human movements. The displays were created by videotaping actors (1 woman and 1 man) who performed various movements of everyday life, including standing-up from or sitting on a chair, crouching, moving the arms/legs in isolation or in combination, touching the floor from a sitted position, stepping aside, and tilting forward or backward. Specifically, the displacements of 22 markers (15 mm in diameter) taped onto major body parts (top of the head, acromions, elbows, wrists, metacarpophalangeal joint of the index fingers, manubrium, xiphoid process, navel, hips, greater trochanters, knees, ankles, toes) of the actors were sampled at a rate of 120 Hz with a four cameras SMART-E motion analysis system (BTS, Milan, Italy). Accordingly, each actor was depicted by a set of white dots moving against a black background (**Figure 1**).

The recordings took place onboard the French Airbus A300-Zero G (Novespace) during three parabolic flights. In parabolic flight, the aircraft is put into a suborbital trajectory (30 parabolas per flight) that provides free-fall. Each parabola includes a pull-up phase and a pull-out phase, each 20–22 s long, where occupants are subjected to around 1.75 times the force of gravity, and a microgravity phase in the middle that lasts about 20 s where gravity is close to zero (0.02 ± 0.018 g). Each parabola is followed by 2 min of normogravity (1 g). Therefore, we recorded the actors’ movements during the microgravity and normogravity phases. In the microgravity phase, the actors always had at least one foot attached on the floor of the aircraft, so that they were not free-floating. This ensured that form characteristics of the point-light displays were comparable under both microgravity and normogravity for similar movements. Furthermore, the starting positions of the different movements depicted in the displays were standardized, the actors having either executed the movement starting from a standing upright position or sitting on a chair. Besides, the viewpoints could differ from one point-light display to another depending on the movement performed and each point-light display was presented in two different viewpoints to increase the number of stimuli. The set of stimuli was composed of 84 point-light displays in total, including 42 normogravity displays and 42 microgravity displays. The 42 displays per condition corresponded to 21 different movements multiplied by the two viewpoints.

### Experimental Design

The participants, lying inside the fMRI scanner, had to watch the point-light displays and indicate whether the movements were performed under normogravity or microgravity (**Figure 1**). The task consisted of three runs of 84 trials each. Each experimental run lasted approximately 11 min. A trial included a point-light display (3 s), followed by an instruction display asking whether the movement was performed on Earth (i.e., normogravity) or in Space (i.e., microgravity). The participants had 2 s maximum to respond as accurately as possible with either of two buttons on a keyboard corresponding to a green or red rectangle on the screen, with the former rectangle coding for a movement performed on Earth and the latter rectangle for a movement performed in Space. A fixation cross was then displayed during the inter-trial interval for an average of 3 s (range 1–12 s), obtained from exponential distribution (Hagberg et al., 2001). The order of the point light displays, including 42 normogravity and 42 microgravity displays per run, and the left-right locations of the rectangles on the screen were randomized across participants and across the three runs.

Stimuli were back-projected onto a semiopaque screen placed at the back end of the MRI tunnel. Participants viewed the displays through tilted mirrors placed over their eyes. They responded with their right index and middle fingers using an MRI-compatible response box. Responses (accuracy) were recorded using a custom software developed using LabVIEW (National Instruments, Austin, TX, United States). Before scanning, the participants had been instructed and had performed a few practice trials on a computer outside the scanning room to ensure understanding of the task.

### fMRI Data Acquisition

The experiment was performed using a 3-T fMRI scanner (Medspec 30/80 AVANCE, Bruker, Ettlingen, Germany). EPI BOLD images were acquired over the three runs (i.e., three fMRI time series) with a T2^∗^ weighted gradient echo-planar imaging sequence [repetition time: 2133.3 ms; echo time: 30 ms; flip angle: 79.5°; 3 mm isotropic voxel size; reco matrix: 64 × 64; 32 interleaved axial slices with 1 mm gap; field of view (FOV): 192 mm × 192 mm]. The scanning planes were parallel to the anterior commissure/posterior commissure and covered the whole brain from the top of the cortex down to the base of the cerebellum. Structural MRI data was acquired using a standard T1-weighted scanning sequence of 1 mm^3^ resolution (MPRAGE; repetition time: 9.4 ms; echo time: 4.424 ms; inversion time: 800 ms; FOV: 256 mm × 256 mm × 180 mm, reco matrix: 256 × 256 × 180) to allow anatomical localization of brain activation.

### fMRI Data Preprocessing and Analysis

Data preprocessing was conducted following the standard SPM8 (Wellcome Department of Imaging Neuroscience, London, United Kingdom^1^) workflow for fMRI (Friston et al., 1995; Henson et al., 1999). Each run consisted of 317 scans, including six dummy images of magnetic field saturation that were discarded before analysis. The remaining images were slice-time corrected. After discarding the last two volumes, these images were realigned to the first image of the time series (6-parameter rigid body) to correct for head movement between scans, and a mean realigned image was created. The realigned images were also “unwarped” to reduce residual movement-related variance (Andersson et al., 2001). Each structural MRI was co-registered to the corresponding mean realigned image, and normalized to a template in the stereotactic space of the Montreal Neurological Institute (MNI) by matching gray matter with a priori gray matter template (Ashburner and Friston, 2005). Normalization was then applied to the functional images before smoothing with a 6 mm × 6 mm × 6 mm Gaussian kernel. The absence of gross normalization errors was visually confirmed by an experienced operator for all participants. No excessive head motion were observed (i.e., cumulative translation and rotation <3 mm and 3° and mean point-to-point translation and rotation <0.15 mm and 0.1°).

Statistical analysis of the fMRI time series was based on the general linear model (GLM) approach (Friston et al., 1994, 1995). The GLM design matrix included the two gravity conditions (i.e., normogravity and microgravity), the instruction, and the fixation cross, which were modeled as boxcar regressors and were convolved with the canonical hemodynamic response function of SPM8. Furthermore, the design matrix also included the participant’s realignment parameters, to regress out residual movement-related variance. Low-frequency drifts were removed from images using high-pass filtering (1/128 Hz). Contrasts of interest were defined at the first level of analysis (i.e., participant-level) to reveal areas coding for: (i) the perception of human movement in point-light displays (i.e., voxels where parameter estimates of the normogravity and microgravity regressors were significantly greater than baseline; labeled “normogravity > baseline” and “microgravity > baseline”); and (ii) gravity information embedded in the displays (i.e., voxels where parameter estimate of the normogravity regressor was significantly greater than that of the microgravity regressor, and inversely; labeled “normogravity > microgravity” and “microgravity > normogravity”). For contrasts (i), active voxels common to both normogravity and microgravity effects were identified by using the contrast “microgravity > baseline” as an inclusive mask of the contrast “normogravity > baseline.” Exclusive masking was also conducted to reveal areas that might have been specifically activated by normogravity or microgravity, although results were insignificant (see Results section). Contrasts (ii), i.e., “normogravity > microgravity” and “microgravity > normogravity,” were masked inclusively with the contrast “normogravity > baseline” to discard any voxel whose activation was unrelated to gravity information (i.e., voxel that can be considered as false positive). Individual contrast maps were then entered into a second level (random effect) full group GLM. It is worth emphasizing that identical results were obtained when implicit baseline (zero) was replaced by the weight of the regressor modeling the fixation periods (see Results section). With respect to group-level analyses, multiple comparisons correction of statistical maps was conducted using a cluster-based extent thresholding for *p* < 0.05 (FWER-corrected) calculated based on the Gaussian random field method and following previous recommendations (Woo et al., 2014).

## Results

All participants were successful in performing the categorization task inside the scanner, with a good and similar categorization accuracy for both the normogravity and the microgravity displays as assessed using independent two-sample *t*-test (mean ± SD: 75.85 ± 7.55% and 72.98 ± 8.22%, respectively; *t* = 1.15; *p* = 0.25; *d* = 0.36).

**FIGURE 2:**
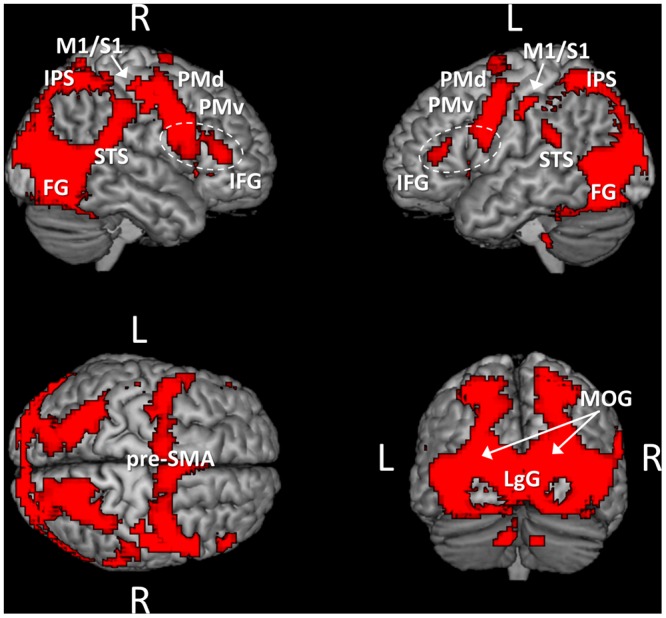
Brain areas activated by the point light displays independently of the gravity condition. The activation pattern was obtained by masking inclusively (*p* = 0.05) the contrast “microgravity > baseline” with the contrast “normogravity > baseline.” Activations are thresholded at *p* < 0.001 (uncorrected) at the voxel level and at *p* < 0.05 (FWE-corrected) at the cluster level. Abbreviations: M1/S1, primary motor and somatosensory cortices; PMd, dorsal premotor cortex; PMv, ventral premotor cortex; pre-SMA, pre-supplementary motor area; IFG, inferior frontal gyrus; IPS, intraparietal sulcus; STS, superior temporal sulcus; FG, fusiform gyrus; LgG, lingual gyrus; MOG, middle occipital gyrus.

As previously mentioned, a first analysis of the fMRI data consisted in identifying brain regions that were similarly activated during the observation of point light displays independently of whether the movements were performed under normogravity or microgravity. For this purpose, we identified voxels that were common to the contrasts “normogravity > baseline” and “microgravity > baseline,” by masking inclusively (*p* = 0.05) the latter contrast with the former contrast (**Figure 2** and **Table 1**). Results indicated that the observation of point light displays moving either under normogravity or microgravity led to a widespread pattern of activity, with significant clusters of activation located in frontal, parietal and occipito-temporal regions. In particular, regions subtending the pattern of activity included the middle occipital gyrus, the lingual gyrus, the fusiform gyrus, the superior, middle and inferior temporal gyrus, the cuneus, the inferior and superior parietal lobules, motor-related areas (primary motor cortex, primary somatosensory cortex, pre-motor cortex, and pre-supplementary motor area) and the inferior frontal gyrus. On the other hand, neither the “normogravity > baseline” contrast nor the “microgravity > baseline” contrast revealed exclusive clusters of significant activation, as examined by looking for activated voxels in either contrasts while using an exclusive masking approach (i.e., “normogravity > baseline” masked exclusively by “microgravity > baseline,” and inversely). Therefore, the networks subtending the perception of human movement under either microgravity or normogravity perfectly overlapped. Furthermore, the same network was identified when baseline was modeled as the fixation period. Significant clusters of activation were located along the regions previously identified, although the spatial extent of activation was reduced (Supplementary Figure S1).

**Table 1:** Activated brain regions during the observation of point light displays, as obtained by masking inclusively (*p* = 0.05) the contrast “microgravity > baseline” with the contrast “normogravity > baseline.”

Region	BA	Side	*X, Y, Z*	*Z*
**Cluster #1: 6082 voxels**				
Middle occipital gyrus	18/19	R	30, -87, 12	>8
			30, -84, 21	>8
		L	–30, -93, 12	>8
			–30, -87, 12	>8
			–27, -90, 15	>8
Fusiform gyrus	19	R	27, -75, -12	7.79
		L	–21, -81, -12	>8
Lingual gyrus	18	R	9, -87, -6	6.28
		L	–6, -87, -9	7.33
Superior temporal gyrus	22	R	60, -36, 21	7.69
		L	–62, -37, 20	6.06
Middle temporal gyrus	37	R	48, -63, 3	>8
		L	–42, -66, 6	>8
Inferior temporal gyrus	19	R	48, -62, -4	>8
		L	–45, -72, 0	>8
Cuneus	17	R	12, -96, 3	7.82
		L	–15, -96, 6	>8
Inferior parietal lobule	40	R	60, -35, 21	7.69
		L	–57, -38, 24	5.93
Superior parietal lobule	7	R	33, -60, 54	7.65
		L	–27, -57, 54	6.78
**Cluster #2: 2069 voxels**				
M1/S1	4	R	51, -12, 39	6.58
		L	–46, -14, 49	6.32
	3	R	54, -9, 48	5.72
		L	–50, -18, 40	5.05
PMd	6	R	39, -3, 51	6.40
		L	–27, -6, 48	7.25
PMv	6	L	–48, 3, 33	6.57
			–42, -3, 42	6.20
			–48, 0, 48	5.69
		R	33, -9, 48	6.33
			48, 0, 45	5.94
Pre-SMA	6	R	9, 12, 51	6.57
			6, 9, 54	6.28
			9, 0, 66	5.12
		L	–3, 9, 51	6.34
			–6, 6, 54	6.32
			–12, -6, 69	4.78
Insula	13	R	30, 24, 0	5.55
		L	–30, 21, 3	4.91
Inferior frontal gyrus	9	R	45, 3, 33	7.37
		L	–48, 6, 27	7.37
	46	R	51, 36, 12	4.79
		L	–45, 33, 15	4.81

*For each region, MNI coordinates at the center of gravity are specified along with the corresponding Brodmann area (BA). *Z*-values refer to significant activation peaks at *p* < 0.001 (uncorrected for multiple comparisons). In addition, all reported regions were significantly active at *p* < 0.05 (FWE correction). Abbreviations: L, left hemisphere; R, Right hemisphere; M1, primary motor cortex; S1, primary somatosensory cortex. PMd, dorsal premotor cortex; PMv, ventral premotor cortex; pre-SMA, pre-supplementary motor area.*

**FIGURE 3:**
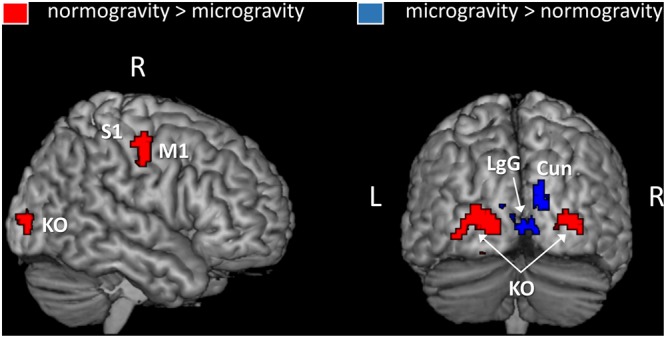
Brain areas showing an activation gradient as a function of the gravity information embedded in the point light displays. Activations are thresholded at *p* < 0.001 (uncorrected) at the voxel level and at *p* < 0.05 (FWE-corrected) at the cluster level. An inclusive mask (*p* = 0.05, “normogravity > baseline” contrast) was applied. Abbreviations: M1, primary motor cortex; S1, primary somatosensory cortex; Cun, Cuneus; LgG, lingual gyrus; KO, kinetic occipital region.

In the second analysis, we identified regions where activation was modulated by the gravity condition of the displays (**Figure 3** and **Table 2**). Three significant clusters of activation were revealed by the contrast normogravity > microgravity. The first cluster was located in the primary motor cortex (BA 4). Activation also extended into the primary somatosensory cortex (BA 3), as shown in greater detail in Supplementary Figure S2. The other clusters belonged to the kinetic occipital region (BA 18) in both the right and left hemispheres. The reverse contrast, namely microgravity > normogravity, revealed increased activation of a cluster that included regions of the right and left lingual gyrus and cuneus.

**Table 2:** Activated brain regions during the observation of normogravity displays vs. microgravity displays and microgravity displays vs. normogravity displays.

Region		BA	Side	*X, Y, Z*	*Z*
**Normogravity** > **Microgravity**					
Middle occipital gyrus		18/19	R	27, -90, 0	4.11
k: 101 voxels			L	–24, -93, 3	4.23
				–27, -83, -3	4.15
				–21, -99, 6	3.46
Motor-related areas (M1/S1)		4	R	51, -12, 39	4.18
k: 45 voxels				56, -8, 48	3.99
		3	R	54, -9, 48	3.98
**Microgravity** > **Normogravity**					
Lingual gyrus		18	R	9, -84, 3	4.01
	k: 107 voxels		L	–9, -78, 0	4.29
Cuneus		17	R	12, -90, 18	3.77
				15, -93, 24	3.63
				18, -84, 18	3.54
			L	–3, -90, 6	3.98

*For each region, MNI coordinates at the center of gravity are specified along with the corresponding Brodmann area (BA). *Z*-values refer to significant activation peaks at *p* < 0.001 (uncorrected for multiple comparisons). In addition, all reported regions were significantly active at *p* < 0.05 (FWE correction). Abbreviations: L, left hemisphere; R, Right hemisphere; M1, primary motor cortex; S1, primary somatosensory cortex.*

## Discussion

The present study investigated whether gravity-related changes in movement kinematics is reflected through activation gradients in regions of the MNS, which would support the premise that coding of gravitational content in BM displays relies on motor resonance. For this purpose, we examined BOLD signal when participants were observing point-light displays of human movements performed under either normogravity or microgravity. The result is twofold: first, independently of the gravity conditions, the perception of human movement relied on a large-scale network that encompassed frontal (inferior frontal gyrus, motor-related areas), parietal (inferior and superior parietal lobules), and occipito-temporal (superior temporal sulcus region, inferior temporal gyrus, fusiform gyrus, lingual gyrus, middle occipital gyrus) regions, which is in keeping with previous findings on the perception of point-light BM (Vaina et al., 2001; Saygin et al., 2004); and second, gravity information modulated the activation of a restricted set of regions of the network including visual (kinetic occipital region, lingual gyrus, cuneus) and motor-related (primary motor and somatosensory cortices) areas. Notably, the portions of the primary motor cortex along with those of the primary somatosensory cortex were significantly more active when acceleration in the point-light displays was consistent with natural (Earth) gravity. Previous studies on the neuronal encoding of the kinematic laws of motion during both abstract (cloud of dots) motion observation (Dayan et al., 2007) and human action observation (Casile et al., 2010) already demonstrated a larger involvement of the motor-related regions in processing normal kinematics compared to perturbed kinematics. The authors proposed that cortical representations of motion are optimally tuned to the kinematic invariants characterizing biological actions, with discrimination of normal vs. abnormal kinematics being carried out via a motor-matching process of the observed movements onto the observer’s motor system. The present finding adds to this view by providing evidence that compliance of gravity cues embedded in the kinematics of human motion with normal gravity is encoded in motor-related areas, possibly by transforming the visual inputs into the specific motor capabilities of the observer and thus coding the plausibility of actually performing the same movements.

Although the above result favors our hypothesis that the interpretation of gravitational cues embedded in BM relies on a mechanism of motor resonance, the primary motor and sensorimotor cortices are not classically considered to be part of the human MNS subtending motor resonance whose core regions are the inferior frontal/ventral premotor and posterior parietal areas (Rizzolatti and Craighero, 2004; Iacoboni and Dapretto, 2006). In particular, several studies that manipulated indirectly the kinematic characteristics of the movement, by contrasting the observation of natural as opposed to unnatural movements (Stevens et al., 2000; Tai et al., 2004; Costantini et al., 2005; Gazzola et al., 2007; Lestou et al., 2008), revealed a further involvement of these core regions in processing movement displays that conform with normal kinematics. However, there is growing evidence that mirror activity extends beyond brain regions identified as being part of the classical MNS, including the primary somatosensory cortex (Keysers and Gazzola, 2009; Molenberghs et al., 2012) and the primary motor cortex (Fadiga et al., 2005; Tkach et al., 2007; Dushanova and Donoghue, 2010). Thus, it is reasonable to assume that the individuals discriminated between normogravity and microgravity displays via the mirror property of these two regions, by simulating the motor commands and their sensory consequences for observed movements.

Such an implicit coding of gravity effects through motor resonance contrasts with a recent study by Maffei et al. (2015) where judgment on graviceptive information embedded in point-light BM is proposed to result from a predictive code generated by an internal model of gravity effects (whose primary sites are in temporo-parietal junction and insula) that is conveyed to the occipito-temporal cortex where it is compared to the incoming stimuli to produce a prediction error, and thereby an activation. Specifically, BM stimuli under a condition of abnormal gravity evoked stronger activation in occipito-temporal regions than BM stimuli under normal gravity. Studies on the recognition of BM when stimuli are displayed upside down (i.e., violation of physical gravity) reconcile this result with our own. Indeed, it was reported a sensitivity to the inversion effect in the occipito-temporal cortex (Grezes et al., 2001; Grossman and Blake, 2001; Pavlova et al., 2004; Peuskens et al., 2005) as well as in parietal (i.e., intraparietal sulcus) and frontal (i.e., caudal part of the middle/inferior frontal gyrus) areas (Grezes et al., 2001; Pavlova et al., 2004) that belong to the MNS. The discrepancy between results from Maffei et al. (2015) and ours may have to do with differences in the complexity of the portrayed movements. They used gait movements that are likely to be of lower complexity than the movements used in our experiment. Exploring gait movements and complex movements close to those used in our experiment, Jastorff and Orban (2009) showed enhanced activations by complex biological kinematics in the occipito-temporal cortex and in frontal regions belonging to the MNS, therefore suggesting that one destination of the BM signals is the occipito-temporal cortex and another destination is the MNS. Accordingly, gravity cues are likely coded in these two main loci of BM processing, with a hierarchy from the occipito-temporal cortex to the MNS as BM becomes more complex.

Another intriguing result was that gravity discrimination between displays also relied on visual regions known to be involved in form-from-motion perception, defined as the ability to extract the form of a stimuli entirely from motion cues. The kinetic occipital region, which was found to be more active for movements performed under normogravity, is selective to kinetic boundaries (Dupont et al., 1997; Van Oostende et al., 1997). In the case of point-light BM, Vaina et al. (2001) showed that this region integrates local motion of the light points with the goal of determining whether they altogether constitute the outline of a human silhouette. The lingual gyrus at the cuneus border, which was inversely found to be more active for movements performed under microgravity, is also involved in processing motion and deriving global form information in the perception of BM (Servos et al., 2002). Accordingly, variations of gravity information in point-light displays of human movement was likely also coded based on the familiarity of the human form reconstructed from the moving dots. Furthermore, the opposite pattern of activation found between the kinetic occipital region (i.e., more active in normogravity) and the lingual gyrus/cuneus complex (i.e., more active in microgravity) may indicate different functions in form-from-motion perception, the former region coding visual familiarity with the observed form-from-motion and the latter region visual unfamiliarity.

In sum, findings of the present experiment suggest that discrimination of point-light movements whose kinematic characteristics either did or did not comply with natural gravity was carried out by (i) mapping the movements onto the observer’s motor system, and (ii) extracting the overall form of the movements from local motion of the moving light points. Such a dual-mechanism plausibly coded both the possibility of actually performing the same movements and the visual familiarity of the observer with the form defined by the movements. Therefore, judgment on graviceptive information embedded in point-light BM may not be restricted to accessing the internal model of gravity effects (Maffei et al., 2015), also relying on motor representations and visual knowledge of what is observed.

## Author Contributions

Conceived and designed the experiment: P-YC, CS, MV, and CA. Subject recruitment and screening: P-YC, MV, and CA. Acquisition of data: FC, P-YC, CS, MV, and CA. Analysis and interpretation of data: FC, P-YC, JM, J-LA, MV, and CA. Drafting the work: FC, MV, and CA. Final approval of the work: FC, P-YC, J-LA, JM, CS, MV, and CA. Being accountable for the accuracy and integrity of the work: FC, P-YC, J-LA, JM, CS, MV, and CA.

## Conflict of Interest Statement

The authors declare that the research was conducted in the absence of any commercial or financial relationships that could be construed as a potential conflict of interest.

## Footnotes

1 http://www.fil.ion.ucl.ac.uk/spm/software/spm8
