# Supplementary material for: Gravity Cues Embedded in the Kinematics of Human Motion Are Detected in Form-from-Motion Areas of the Visual System and in Motor-Related Areas
Source: Front Psychol. 2017 Aug 17;8:1396. doi: 10.3389/fpsyg.2017.01396 (PMC5562714; doi:10.3389/fpsyg.2017.01396)
Supplement: Supplementary file 1 [file Data_Sheet_1.DOCX]

**Supplementary material**

**Figure S1.** Brain areas activated by the point light displays. The activation patterns were obtained by intersecting either the contrasts “normogravity>baseline” and “microgravity>baseline” (panel a) or the contrasts “normogravity>fixation” and “microgravity>fixation” (panel b). Activations are thresholded at p < 0.001 (uncorrected) at the voxel level and at p < 0.05 (FWE-corrected) at the cluster level. Abbreviations: M1/S1, primary motor and somatosensory cortices; PMd, dorsal premotor cortex; PMv, ventral premotor cortex; pre-SMA, pre-supplementary motor area; IFG, inferior frontal gyrus; IPS, intraparietal sulcus; STS, superior temporal sulcus; FG, fusiform gyrus; LgG, lingual gyrus; MOG, middle occipital gyrus.

**Figure S2.** Cluster in the primary motor cortex showing higher activation during the observation of normogravity displays versus microgravity displays (labelled 1G>0G). Masks of the primary motor (M1) and somatosensory (S1) cortices have been reported to help in locating the cluster activation foci. Masks were obtained using WFU PickAtlas toolbox available at [www.ansir.wfubmc.edu](http://www.ansir.wfubmc.edu) (Lancaster et al., 2000; Maldjian et al., 2003).

Lancaster JL, Woldorff MG, Parsons LM, Liotti M, Freitas CS, Rainey L, et al. (2000) Automated Talairach atlas labels for functional brain mapping. *Hum Brain Mapp* 10: 120-131.

Maldjian JA, Laurienti PJ, Burdette JB, Kraft RA (2003) An Automated Method for Neuroanatomic and Cytoarchitectonic Atlas-based Interrogation of fMRI Data Sets. *NeuroImage* 19: 1233-1239.
