# Supplementary figures and images for: Gravity Cues Embedded in the Kinematics of Human Motion Are Detected in Form-from-Motion Areas of the Visual System and in Motor-Related Areas
Source: Front Psychol. 2017 Aug 17;8:1396. doi: 10.3389/fpsyg.2017.01396 (PMC5562714; doi:10.3389/fpsyg.2017.01396)

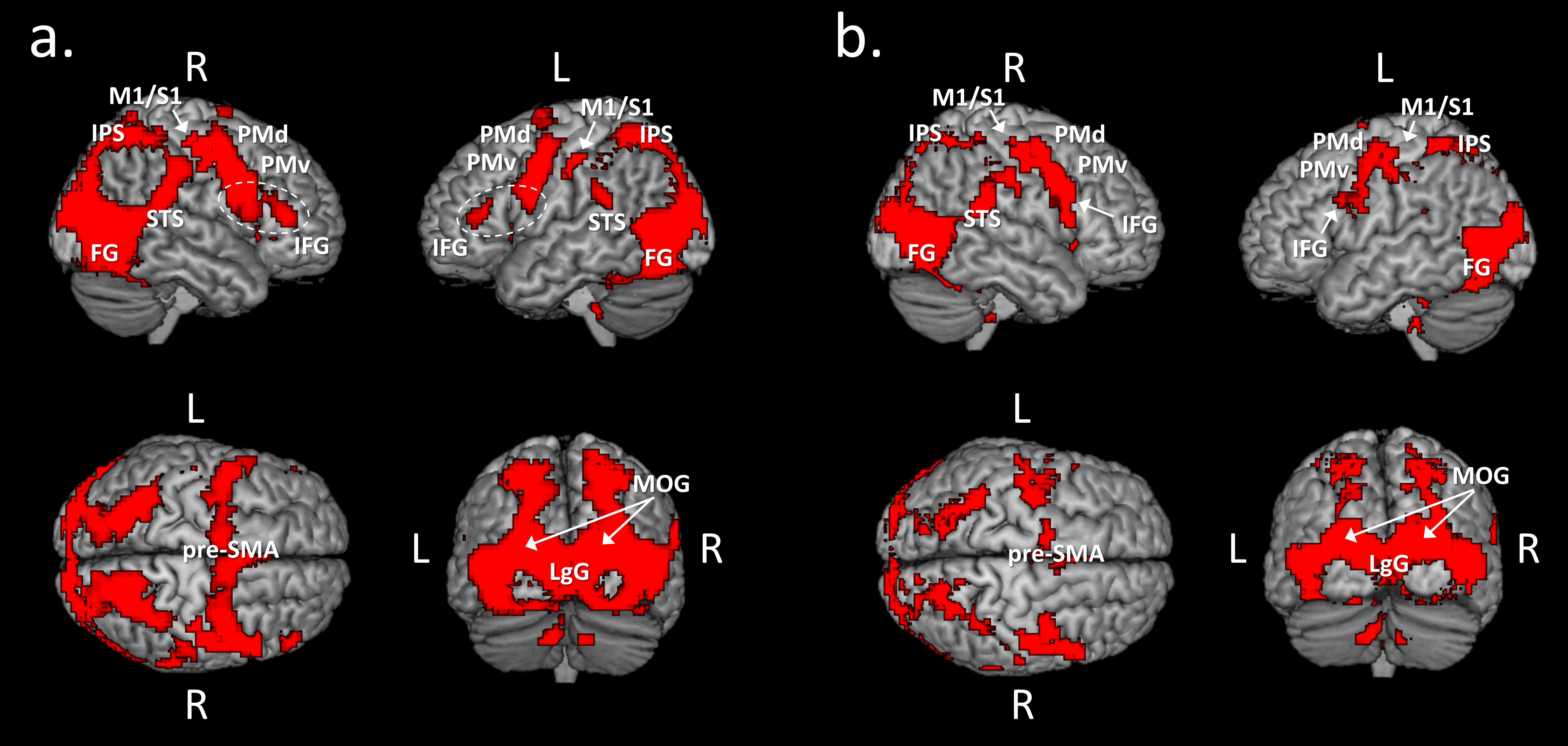

Supplement: Supplementary file 2 [file Image_1.TIF]

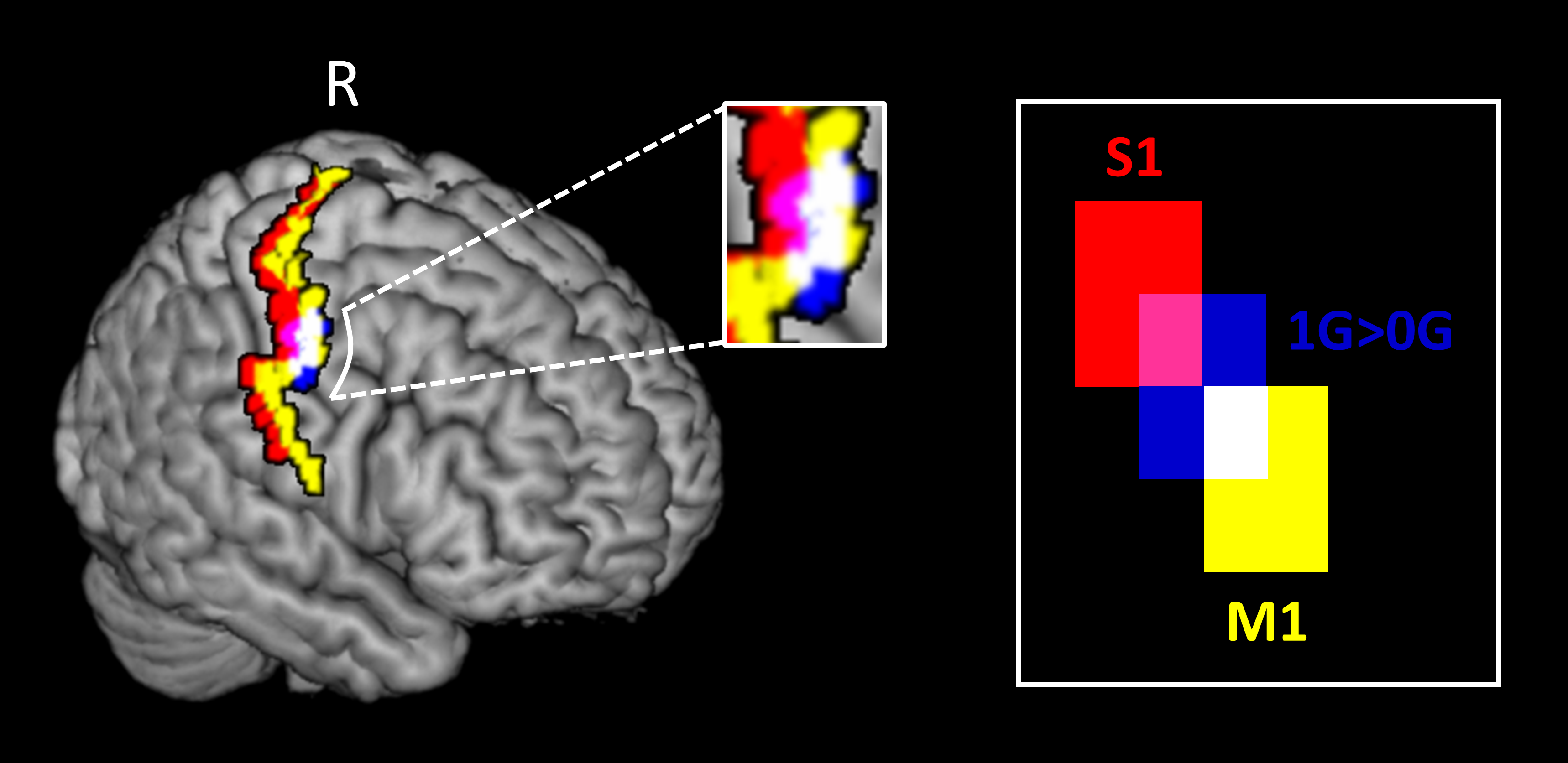

Supplement: Supplementary file 3 [file Image_2.TIF]
